# The Association between Oxytocin and Social Capital

**DOI:** 10.1371/journal.pone.0052018

**Published:** 2012-12-19

**Authors:** Takeo Fujiwara, Laura D. Kubzansky, Kenji Matsumoto, Ichiro Kawachi

**Affiliations:** 1 Department of Social Medicine, National Research Institute for Child Health and Development, Tokyo, Japan; 2 Department of Society, Human Development, and Health, Harvard School of Public Health, Massachusetts, United States of America; 3 Department of Allergy and Immunology, National Research Institute for Child Health and Development, Tokyo, Japan; Umeå University, Sweden

## Abstract

**Background:**

Oxytocin is known to be related to social behaviors, including trust. However, few studies have investigated the association between oxytocin levels and social capital. Thus, we tested the hypothesis that endogenous oxytocin levels are positively associated with social capital. We also considered whether the association differed across gender because previous studies have shown differential effects of OT on social behaviors depending on gender.

**Methods:**

We recruited a convenience sample of 50 women and 31 men in Japan via community sampling from whom we obtained urine sample with which to measure oxytocin levels. Individual-level cognitive social capital (social trust and mutual aid) and structural social capital (community participation) were assessed using a questionnaire. We used multivariate regression, adjusted for covariates (age, number of children, self-rated health, and education), and stratified by gender to consider associations between oxytocin and social capital.

**Results:**

Among women, oxytocin was inversely associated with social trust and mutual aid (p<0.05). However, women participating in only 1 organization in the community showed higher oxytocin than women who participated in either no organizations (p<0.05) or 2 or more organization (i.e. inverse-U shape association). Among men, no association was observed between oxytocin and either form of cognitive and structural social capital.

**Conclusion:**

Women who perceived low cognitive social capital showed higher oxytocin levels, while structural social capital showed inverse-U shape association with oxytocin. No association between oxytocin and social capital was found among men. Further study is needed to elucidate why oxytocin was inversely associated with cognitive social capital only among women.

## Introduction

Oxytocin (OT), a neuropeptide composed of nine amino acids [1], is known to have both peripheral effects (e.g., uterine contraction during labor and milk ejection during lactation) [2] and central effects (e.g., maternal behavior, pair bonding, and social recognition) [3]. Previous studies employing exogenous OT administration have shown links between OT and social behaviors in both animals [4], [5] and humans [6], [7], and a number of studies have reported associations between endogenous circulating levels of OT and social behaviors. For example, plasma OT levels in early pregnancy and the postpartum period were significantly positively correlated with maternal behaviors towards the offspring (e.g., gaze, affectionate touch, and frequent infant checking) [8]. Also, momentary experiences of romantic love were associated with pulsatile increases in OT levels among heterosexual romantic couples [9]. Further, OT is associated with trust in others: in the “trust game,” which is characterized by sequential, anonymous monetary payoffs, the trustee’s plasma OT level was significantly higher when the trustee received money intentionally than randomly; further, the amount of money returned to the investor was correlated with OT level [10].

According to Fukuyama, trust is defined as the expectation of regular, honest, and cooperative behavior from others that arises within a community; such expectations are based on commonly shared norms [11]. Thus, a property that can be called “social trust” arises from the prevalence of trust in a given community. Social trust is an aspect of social capital that has shown consistent positive effect on self-rated physical health [12], [13], [14], [15], [16], [17], [18], [19], [20], mental health [15], [18], [21], [22], [23], [24], [25], [26], and health behaviors [27], [28]. Social capital is defined as the resources that individuals can access through their social connections to others. The resources available through social networks can take several distinct forms, including: a) levels of trust between individuals, b) reciprocity exchanges, c) ability to undertake coordinated, collective action. For example, a community with high social capital is one in which members frequently exchange favors for one another. These reciprocity exchanges in turn hinge on high levels of interpersonal trust (i.e. trust that a recipient of a good deed will return the favor in the future). Therefore, it is reasonable assumption that OT might be positively associated with individual-level social capital, ie, perception of social trust among neighbors.

Prior research has suggested that, social capital, especially community participation, tends to be higher among women than men [29], [30]. Prior work in humans has often found effects of oxytocin to differ by gender, although levels of OT in urine do not diff by gender [31]. For example, in one study, a significant positive association between warm contact and OT was found among women, but not among men [32]; another study showed that the effects of OT on social behaviors (e.g., negative engagement and interactive stress) differ by gender [31]. Based on prior hints of gender differences in the effects of oxytocin, we hypothesized that a stronger association between social capital and OT would be observed among women than men.

Social capital can be decomposed into two dimensions: cognitive social capital and structural social capital [33]. The cognitive dimensions of social capital includes perceptions of trust as well as beliefs about the extent to which neighbours can be called upon to provide social support, and structural dimensions of social capital refers to reports of actual behaviours, such as participating in locally based associations [34]. These dimensions might be differently associated with OT, because the structural dimension requires actual behaviors in order to participate in some associations, while the cognitive dimension does not included actual behaviors. As several factors affect actual behaviors rather than perception per se, we hypothesized that cognitive social capital is more likely to be associated with OT than structural social capital. Therefore, the purpose of this study is to examine the association between circulating OT levels and individual-level social capital, decomposed by cognitive and structural dimensions and stratified by gender.

**Table 1:** Distribution of demographics, oxytocin, and social capital variables by gender.

		Female (n = 50)				Male (n = 31)			
		N or Mean	% or SD	Min	Max	N or Mean	% or SD	Min	Max
Age	Year	35.9	3.9	24	44	36.9	2.8	31	42
Number of children	1	26	52.0			18	58.1		
	2	16	32.0			11	35.5		
	3	8	16.0			2	6.5		
Self-rated health	1 (excellent)	20	40.0			14	45.2		
	2 (very good)	19	38.0			10	32.3		
	3 (good)	6	12.0			7	22.6		
	4 (fair)	5	10.0			0	0		
	5 (poor)	0	0.0			0	0		
Education	High school or less	7	14.0			6	19.4		
	Some college	27	54.0			3	9.7		
	College or more	16	32.0			22	71		
Social trust	High	10	20.0			7	22.6		
	Middle	29	58.0			15	48.3		
	Low	11	22.0			9	29		
Mutual aid	High	13	26.0			7	22.6		
	Middle	21	42.0			16	51.6		
	Low	16	32.0			8	25.8		
Community participation	0 organization	15	30.0			11	35.5		
	1 organization	14	28.0			10	32.3		
	2 or more organizations	21	42.0			10	32.3		

## Methods

### Participants

The study was approved by the Ethics Committee of National Institute for Public Health, and all participants signed informed consent forms prior to enrollment in the study. Eighty-one participants (50 women and 31 men [the men were spouses of participating women]) were recruited through personal connections with research coordinators or the participants themselves (i.e., a convenience snowball sample). The eligibility criteria restricted the sample to women and men with 18–48-month-old children, because a previous study reported that OT levels were stable among women and men with children [31].

**Table 2:** Summary of oxytocin measurements.

	Mean	SD	Median	Min	Max
Female (n = 50)	108.9	38.2	98.1	53.5	227.8
Male (n = 31)	112.8	42.1	109.3	54.0	236.2

unit: µU/ml per creatinin g/L.

### Procedure

Research coordinators visited the participants’ home for approximately 1 hour between 11 a.m. and 2 p.m. A questionnaire was sent before the home visit and was collected during the home visit. We collected urine samples, because they are noninvasive and easy to collect and assay. In animal models, urinary oxytocin assays have been reported to provide a valid biomarker [35]. A 1-mL urine sample was collected in a tube to which a 40-µl aliquot of sodium citrate buffer (0.03 M sodium citrate, 25 mM EDTA and 0.35 mM 1,10-phenanthroline) was added; samples were immediately kept in a cooler box at 4°C for maximum of 2 hours and kept at −20°C in the laboratory.

**Figure 1:**
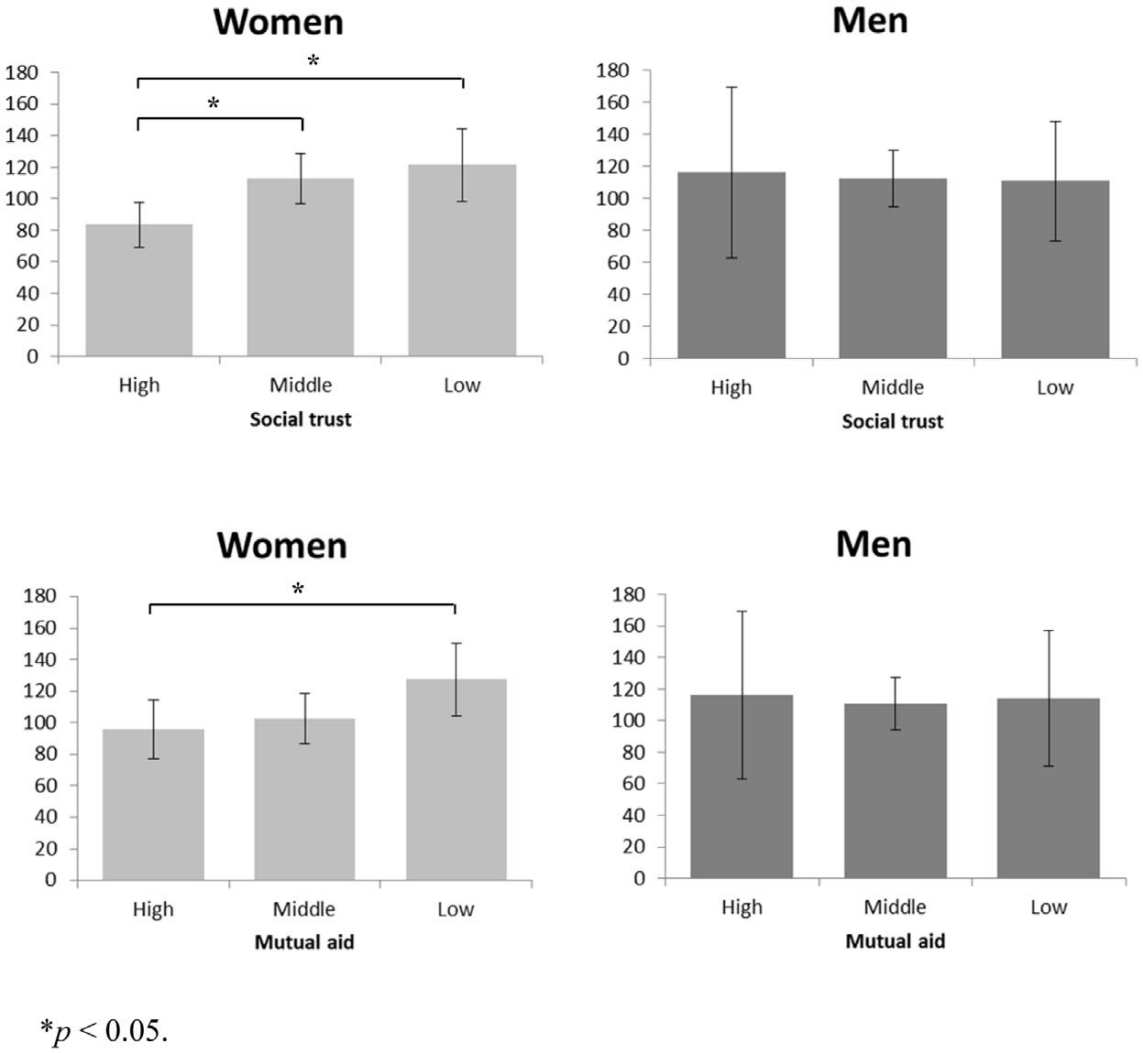
Association between social capital and urine oxytocin by gender.

### Oxytocin Analysis

OT concentrations in the urine samples were measured by a competitive radioimmunoassay, as described elsewhere [36]. The radioimmunoassay method to measure urinary OT per mg creatinine showed a strong correlation with plasma OT measurements in a previous study (r  = 0.89) [37]. Briefly, rabbit antiserum specific for human OT was generated by immunizing a rabbit four times with recombinant human OT (ASKA Pharmaceutical. Co., Ltd., Tokyo, Japan) conjugated with water-soluble carbodiimide (Nakarai Tesque, Tokyo, Japan). The urine sample was decomplimented at 56°C for 30 min, and the supernatant was extracted after centrifugation (3,000 rpm, 10 min, 4°C). The decomplimented sample and the same amount of ^125^I-labeled OT (Perkin Elmer Life Sciences, Inc., Boston MA) solution were allocated for use in an assay tube (Shionogi, Tokyo, Japan). Then, rabbit anti-OT serum was added to each assay tube, followed by incubation for 2 days at 4°C. Next, goat anti-rabbit IgG serum (ASKA Pharmaceutical. Co., Ltd., Tokyo, Japan) was added to each assay tube, followed by incubation for one day at 4°C. After centrifugation, the radioreactivity of the pellet was measured by a gamma counter (Auto Well Gamma System ARC-1000 M, Aloka, Tokyo Japan). According to the standard curve, the minimal detection limit of this assay was 3 µU/ml (1 µU of OT is equivalent to 1.776 pg). All assays were performed in duplicate. The assay’s intra- and inter-assay coefficients of variability were <14.2%. The concentration of OT in the urine was standardized according to the urinary creatinine concentration. Urinary creatinine was measured using the alkaline picrate colorimetric method (modified Jaffe).

**Table 3:** Association between urine oxytocin and social capital indicators by gender.

		Female (n = 50)					Male (n = 31)					P for interaction between gender and social capital variables*
			Crude		Adjusted*			Crude		Adjusted*		
		Mean OT level (SD)unit: µU/ml per creatinin g/L	B	95% CI	B	95% CI	Mean OT level (SD) unit: µU/ml per creatinin g/L	B	95% CI	B	95% CI	
Cognitive social capital												
Social trust	High	83.4 (19.7)	ref		ref		116.2 (57.7)	ref		ref		0.29
	Middle	112.9 (41.3)	**29.5**	**(2.5 to 56.5)**	**29.0**	**(1.4 to 56.5)**	112.4 (31.6)	−3.8	(−44.6 to 37.0)	−2.1	(−46.8 to 42.7)	
	Low	121.3 (34.2)	**37.8**	**(5.7 to 70.0)**	**33.5**	**(0.3 to 66.7)**	110.9 (48.9)	−5.3	(−5.3 to 39.6)	−7.9	(−62.2 to 46.4)	
	P for trend		**0.024**		**0.046**			0.81		0.77		
Mutual aid	High	95.9 (31.0)	ref		ref		116.2 (57.7)	ref		ref		0.38
	Middle	102.7 (34.5)	6.8	(−19.2 to 32.8)	4.8	(−22.0 to 31.6)	110.7 (31.2)	−5.5	(−45.8 to 34.9)	−3.1	(−47.8 to 41.6)	
	Low	127.5 (42.9)	**31.6**	**(4.1 to 59.1)**	28.7	(−0.6 to 57.9)	114.1 (51.2)	−2.1	(−48.2 to 44.0)	−5.5	(−58.8 to 47.8)	
	P for trend		**0.021**		**0.046**			0.94		0.83		
Structural social capital												
Community participation	2 or more	103.7 (33.8)	2.3	(−23.3 to 27.9)	12.0	(−16.1 to 40.1)	106.8 (32.9)	−18.4	(−56.4 to 19.7)	−17.7	(−57.3 to 21.9)	0.34
	1	124.5 (45.5)	23.1	(−5.0 to 51.3)	**29.8**	**(1.1 to 58.6)**	105.2 (44.7)	−20.0	(−58.0 to 18.0)	−39.0	(−89.7 to 11.6)	
	0	101.4 (34.9)	ref		ref		125.2 (47.7)	ref		ref		
	P for trend		0.99		0.52			0.32		0.35		

* Adjusted for age, number of children, self-rated health, and education.

### Assessment of Social Capital

Individual perceptions of community social capital were assessed within both cognitive and structural domains, following the approach most widely practiced in the previous literature [29], [30], [38], [39]. Indicators of cognitive social capital included items measuring social trust and mutual aid. Social trust was assessed with a single item: “Do you think that people in your neighborhood trust each other?” with 4-Likert-scale responses of “yes”, “somewhat yes”, “somewhat no”, and “no”. This measurement of social trust is somewhat different from standard measures, such as “In general, would you say that your neighbors can be trusted?” [40]. However, by asking only about whether the individual believe neighbors can be trusted, the latter question cannot measure perceived community norms in terms of mutual trust because it captures only an individual’s perception of the trustworthiness of his or her neighbors and not how trustworthy neighbors might perceive each other to be [34], [41]. Thus, we used a different question to measure the perceived norms of social trust in the community, which has also been used in previous studies [29], [30], [38], [39]. The responses “relatively no” and “no” were collapsed a priori in order to create three categories, as the “no” group was too small (4.3%) by itself to permit further statistical analysis; thus, the response distribution across categories was high trust (20.4%), middle trust (53.8%), and low trust (25.8%). Mutual aid was assessed with a single item: “Do you think that people in your neighborhood aid each other?” with 4-poin Likert-scale responses of “yes”, “somewhat yes”, “somewhat no”, and “no” as used in previous studies [29], [38], [39]. The responses “somewhat no” and “no” were collapsed a priori in order to create three categories, as the “no” group was too small (6.5%) by itself to permit further statistical analysis; thus, the response distribution across categories was high mutual aid (23.7%), middle mutual aid (45.2%), and low mutual aid (31.2%). The distribution of responses were quite similar with those obtained in a previous study using a community representative sample of middle-aged women in Japan [39].

Structural social capital was assessed by asking the participants about their community participation, which was calculated as the number of organizations in which the respondent reported participating; this measure has been used in previous studies to show the association with health [39], [42]. Organizations with which participants reported being involved included child rearing circles, parent-teacher associations, civic organizations, consumers’ cooperative societies, unions/religious groups, or other community groups. On the basis of the distribution of responses, we categorized community participation into three groups: no participation (38.3%), 1 organization (27.7%), and 2 or more organizations (34.0%). Community participation was lower in our sample than in a previous study using a community representative sample of middle-aged individuals in Japan (no participation [23.5%], 1 organization [23.2%], and 2 or more organizations [53.4%]) [39], probably because our sample had younger children.

### Covariates

Covariates include age, number of children, self-rated health (5-point Likert scale), and education (high school or less, some college, and college or more). All were measured via questionnaire.

### Statistical Analysis

Associations between cognitive and structural social capital and urinary OT level were analyzed using regression models stratified by gender. First, bivariate models were performed to determine crude association. Then, multivariate models were performed with adjustment for age, number of children, self-rated health (5-point Likert scale), and education (high school or less, some college, and college or more) to see the independent association between social capital and OT. Further, we tested for an interaction effect between gender and social capital on OT. All analyses were performed using the STATA MP version 12.0 software package (STATA Corporation, College Station, TX, 2011).

## Results

Table 1 shows the distribution of demographic and social capital indicators among participants by gender. Basically, participants were middle-aged (mean for women: 35.9 [SD: 3.9] years, men: 36.9 [SD: 2.8] years), more than 90% of them reported their health as being good or better, and the majority graduated high school. Levels of social trust were higher among women than men: 22% of women reported low social trust, while 29% of men reported the same. Higher community participation was observed among women than men (i.e., 42% and 32% of them participated 2 or more organization, respectively).

OT levels for both men and women are described in Table 2. The mean (SD) OT levels were similar between women and men: 108.9 (38.2) and 112.8 (42.1) µU/mL per creatinine g/L, respectively; t-test indicated these results were not significantly different (p>0.6).

Table 3 and Figure 1 show the association between OT and social capital indicators. Among women, an inverse association between social trust and OT level was observed: mean OT level of women who perceived high, middle, and low social trust were 83.4, 112.9, and 121.3 µU/mL per creatinine g/L, respectively, showing a significant inverse dose-response association (p for trend  = 0.024). This association remained significant even after adjustment for covariates (age, number of children, self-rated health, and education, p  = 0.046). Similarly, women who perceived low mutual aid showed higher OT level than those who perceived mutual aid as middle or high (p  = 0.021); these effects remained significant in the adjusted model (p  = 0.046). However, the association between OT and structural social capital (i.e., community participation) was not linear: the mean OT levels of women who participated in 2 or more, 1, or 0 organizations were 103.7, 124.5, or 101.4 µU/mL per creatinine g/L, respectively, suggesting an inverse-U shape association. In the adjusted model, women who participated in 1 organization showed OT levels 29.8 µU/mL per creatinine g/L higher than women who did not participate in any organization, a significant effect (p  = 0.042). By contrast, no association was found between OT and social capital indicators among men (p>0.3).

Further, the interaction effect of gender and social capital on OT was considered. However, we found no interaction effect between gender and social capital on OT (p>0.2, Table 2), probably due to small sample size.

## Discussion

The current study revealed that among women, OT levels measured in the urine –a proxy of circulating peripheral OT level–was inversely associated with cognitive social capital: those perceiving lower social trust showed higher OT levels than those perceiving high social trust. Interestingly, OT level showed an inverse U-shaped association with structural social capital, with women participating in 1 organization showing higher OT levels than women participating in either 0 or 2 or more organizations. Among men, no association was observed between OT and indicators of social capital. However, because of the study’s cross-sectional design, our findings cannot provide insight into the causal direction of effects; only an association between OT and social capital can be confirmed.

To the best of our knowledge, this is the first study that has considered the association between OT levels and social capital. Previous studies have reported that baseline plasma OT was positively associated with relationship stress among young women [43] but inversely associated with frequency of social contacts among postmenopausal women [44]. Thus, it is possible that exposure to a community with low social trust or mutual aid induces relationship stress or less frequent contact with neighbors, resulting in elevations in circulating OT levels. It has also been reported that smaller social networks lead to higher levels of stress biomarkers (e.g., cortisol [45]) and inflammation marker (e.g., IL6-receptor and C-reactive protein [46]), although social networks and social capital are not exactly the same.

Our study showed an inverse association between OT and social trust, although other studies reported positive association between plasma OT and general trust [6], [10]. One possible explanation for this discrepancy is that urinary and plasma OT levels might show different effects of trust. For example, Feldman et al. reported that urinary OT was associated with interactive stress, while plasma OT was associated with affect synchrony [31]. Another possible reason for this difference might be that we investigated the general concept of trust between neighbors without directly asking whether the respondents trust any particular neighbor. In other words, we measured the respondents’ perception of community, which they could interpret as a stressor, not as a proxy of their tendencies towards trust in others (i.e. general trust). Women who perceived low social trust might have higher levels of community-related stressors, and chronic exposure to such stress from community sources might elevate circulating OT levels, as indicated by other studies [43], [44]. Alternatively, women who perceived low social capital within their community might be more likely spend time with their families to avoid contacts with neighbors, which in turn could lead to more positive experiences with parenting or romantic relationships with partner, both of which enhance OT [8], [9].

The reasons for the inverse U-shaped association observed between structural social capital and urinary OT among women are unknown. However, it might be that participating in one organization is a stressor for women–especially during child raising–because the women might be unwilling or uninterested participants in the organization, only participating because of the duty to make friends for their children or receive useful information for child rearing. Women who participate in more organizations may find it to be a positive experience, whereas those who participate in only one may tend to do so out of obligation, therefore finding it less satisfying or more stressful. Further study is needed to replicate and understand the inverse U-shaped association between structural social capital and OT among child-rearing populations in other settings.

Among men, OT was not significantly associated with indicators of social capital, perhaps because of the smaller number of enrolled men than women and resulting lower power to detect effects. However, this result is consistent with those of previous studies, which have found weaker effects of OT on blood pressure and norepinephrine [32]–which are also linked with stress–in men. It is possible that even if men perceived low social capital in the community or participated in community organizations, this is not a stressor for them in the same way as it is for women; this is plausible given other research suggesting that community social capital is less important–and work-place social capital more important–for men than women. A previous study reported that workplace social capital was associated with hypertension among male, but not women [47]. Therefore, it is somewhat less surprising that OT levels did not differ according to perceived social capital among men. Further research is needed to elucidate the association between OT and workplace social capital among men.

Several limitations need to be addressed before drawing firm conclusions. First, the participants were a convenience sample and limited to middle-aged adults with children; this precludes the generalizability of the findings. However, our sample was selected from various locations in greater Tokyo and covers a wide range of social capital and socioeconomic status. Second, we used spot urine samples as a proxy of circulating OT, but urinary OT might be different from plasma OT, saliva OT (i.e. peripheral OT) or cerebrospinal fluid OT (i.e. central OT) [31], [48]. Further study is needed to replicate these findings using other OT measurement in order to determine whether other forms of OT may yield different insights into the underlying biological processes related to trust. Third, our cross-sectional design precludes conclusions about the causal relationship between social capital and OT, that is, whether higher OT causes low social capital or vice versa. Future prospective study is needed to confirm whether living in low-social-capital community elevates circulating OT levels.

In conclusion, we found an inverse association between urinary OT and cognitive social capital among women. Structural social capital showed an inverse U-shaped association with OT among women. Having low social capital might be a stressor for women who are raising children, as shown by their high OT levels. No association was found between OT and social capital among men. Further study using prospective design and other peripheral or central OT measurements is warranted to confirm whether having low social capital elevates levels of circulating OT.
